# A Metal–Organic Hybrid Composed of Dual Quenching Cofactors as a Nanoquencher for the Fluorescent Determination of Protease Caspase-3

**DOI:** 10.3390/bios15060354

**Published:** 2025-06-04

**Authors:** Fengli Gao, Lin Liu, Cancan He, Yong Chang, Weiqiang Wang

**Affiliations:** 1 Henan Province Key Laboratory of New Opto-electronic Functional Materials, College of Chemistry and Chemical Engineering, Anyang Normal University, Anyang 455000, China; flgao@aynu.edu.cn (F.G.); 13403862830@163.com (C.H.); 2 Shiyan Key Laboratory of Biological Resources and Eco-Environmental Protection, Department of Chemistry and Environmental Engineering, Hanjiang Normal University, Shiyan 442000, China; 3 International Joint Laboratory of Henan Photoelectric Functional Materials, College of Chemistry and Chemical Engineering, Anyang Normal University, Anyang 455000, China; 17398688109@163.com

**Keywords:** metal–organic hybrid, protease, nanoquencher, caspase-3, apoptosis

## Abstract

Nanoquenchers with a single quenching cofactor exhibit limited fluorescence quenching efficiency. In this work, a metal–organic hybrid with dual quenching cofactors (Cu^2+^ and pyrroloquinoline quinone or PQQ) was prepared by metal-coordinated assembly and used as a nanoquencher for a protease assay with enhanced quenching efficiency. The peptide substrate with an oligohistidine (His_6_) tag was labeled with a fluorophore. Caspase-3 was determined as a protease example. The substrate was attached onto the surface of the Cu-PQQ nanoquencher by a metal coordination interaction between the unsaturated Cu^2+^ on the nanoparticle surface and the His_6_ tag in the peptide. The cleavage of the peptide substrate by enzymatic hydrolysis led to the release of a fluorophore-conjugated segment from the nanoquencher surface, thus turning on the fluorescence. The nanoprobe was used to determine caspase-3 with a linear range of 0.01–5 ng/mL and a detection limit of 7 pg/mL. Furthermore, the method was used to evaluate inhibition efficiency and monitor drug-induced cell apoptosis. In contrast to other means of peptide immobilization, such as physical adsorption and covalent coupling, the strategy based on the metal coordination interaction is simple and powerful, thereby achieving assays of caspase-3 activity in lysates with a satisfactory result. The work should be valuable for the design of nanoquenchers with multiple quenching cofactors and the development of novel biosensors.

## 1. Introduction

Proteases are important enzymes that can catalyze the hydrolysis of amide bonds in well-defined peptide substrates. Their functions are essential for different physiological processes, including protein catabolism, food digestion, wound healing, and so forth. The activities of proteases are tightly related to a variety of diseases, e.g., inflammation, thrombosis, diabetics, neurodegenerative diseases, and cancers [1,2,3]. Inhibitors against protease-related diseases can be utilized as potential treatment drugs. Enormous efforts have been devoted to developing viable assays for the determination of proteases and the screening of their inhibitors, including Western blotting, fluorescence, surface plasmon resonance, surface-enhanced Raman scattering spectroscopy, colorimetry, electrochemistry, electrochemiluminescence, and photoelectrochemistry [1,2,4,5,6,7,8,9,10]. Among them, the homogeneous fluorescence assay is the most popular method for protease detection due to its advantages of simplicity, sensitivity, rapidness, and reproducibility [11,12,13,14,15]. Generally, the peptide-based fluorescence assay relying on fluorescence resonance energy transfer (FRET) requires the conjugation of a fluorophore and a quencher at the dual terminal ends of the peptide substrate, respectively. The protease-mediated hydrolysis of the peptide will cause a change in the distance between the fluorophore–quencher pair, leading to a decrease in FRET efficiency and the recovery of the initially quenched fluorescence. Despite remarkable achievements, traditional organic dye-based FRET systems suffer from well overlap between the absorption and emission spectrum, and their relatively low energy efficiency (~50%). Therefore, it is still desirable to design novel systems for the quantification of proteases and the evaluation of their activity.

A series of nanomaterials have been exploited as nanoquenchers to quench the emission of fluorophores by different mechanisms, such as carbon nonomaterials, gold nanoparticles, metal–organic hybrids, metallic oxides or dichalcogenides, and so forth [2,16,17,18,19,20,21,22,23]. Among them, metal–organic hybrids, assembled by metal ions and organic ligands through coordination interactions, have attracted widespread attention recently in consideration of their multiple recognition sites and excellent fluorescence quenching ability, including metal–organic frameworks (MOFs), metal–polymer nanoparticles, and metal–ligand coordination nanoparticles [19,24]. Such nanomaterials have been utilized as excellent nanoquenchers for the detection of metal ions, nucleic acid, and proteins [25,26,27,28,29]. For instance, Zhu et al. reported a sensing platform for biomolecules that uses N,N’-bis(2-hydroxy ethyl)dithiooxamidatocopper(II) [Cu(H_2_dtoa)] MOFs to quench the fluorescence of a DNA probe with a quenching efficiency of 84.53% [27]. Yang et al. developed a fluorescent biosensor for the detection of human immunodeficiency virus DNA and RNA sequences by using three-dimensional Cu-based zwitterionic MOFs as the quenchers, with a quenching efficiency ranging from 65% to 76% [28]. Qiu et al. demonstrated that the Cu-MOFs with N-carboxymethyl-(3,5-dicarboxyl)pyridinium bromide and phenanthroline ligands could quench the fluorescence of a DNA probe with a quenching efficiency of 75% [29]. Generally, the DNA probes in the methods were adsorbed on the surface of metal–organic hybrids through the coordination interactions between the unsaturated metal ions and the DNA phosphate backbone as well as the π-π stacking interactions between the organic linkers and the nucleobases. Subsequently, the fluorescence of dyes linked at the DNA probes was quenched by the metal–organic hybrids through an energy or electron transfer effect. However, these metal–organic hybrids with a single metal cofactor as the quencher show limited quenching efficiency, and the bulks suffering from poor dispersion in solution will hinder their applications in vivo [30,31,32]. In addition, the methods for the immobilization of dye-labeled probes on the surface of metal–organic hybrids by covalent coupling or physical adsorption are complex or less stable, and there have been few studies on the utilization of metal–organic hybrids for the fluorescent determination of protease activity [33]. Therefore, the development of novel metal–organic hybrids with multiple quenching cofactors for fluorescence sensing platforms still remains a significant challenge.

**Scheme 1:**
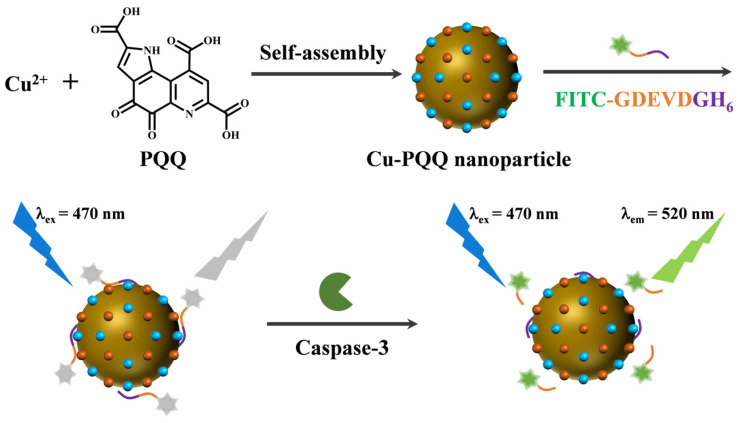
Schematic illustration of the fluorescent biosensor for caspase-3 detection with the Cu-PQQ nanoparticle as the nanoquencher.

Besides the unique properties provided by metal ions themselves, organic ligands in metal–organic hybrids can also endow nanomaterials with certain excellent properties, such as redox, light-responsive, and pH-sensitive activeness [34,35,36,37]. Natural amino acids, polyphenols, and quinones can be used as the ligands of metal–organic hybrids for diverse applications by coordination-driven self-assembly [38,39,40,41]. As a redox cofactor, pyrroloquinoline quinone (PQQ) with an *o*-quinone moiety exhibits a unique redox property as an antioxidant and biocatalyst for the oxidation of thiols [42]. In addition, quinone derivatives including PQQ exhibit good fluorescence quenching abilities [43]. More interestingly, PQQ contains three carboxyl groups that may serve as the binding sites to coordinate metal ions for the construction of metal–organic hybrids [44]. Given that copper ions have a strong fluorescence quenching ability and His-binding properties [45,46,47], the Cu-PQQ metal–organic hybrid was prepared through a typical hydrothermal method and then used as an excellent nanoquencher for the detection of protease (Scheme 1). Caspase-3 was chosen as the model because it plays a key role during the induction and execution phases of apoptosis [48,49]. The peptide substrate was designed with three domains—a cleavage sequence Asp-Glu-Val-Asp (DEVD) specific to caspase-3, a fluorophore (fluorescein isothiocyanate, FITC) linked at the N terminus, and a hexahistidine (His_6_) tag included at the C terminus. The peptide probe can be captured by Cu-PQQ through the metal coordination interaction between the unsaturated Cu^2+^ on the nanoparticle surface and the His_6_ tag in the peptide substrate [46]. The close distance between the fluorophore (FITC) and the dual quenching cofactors (Cu^2+^ and PQQ) leads to the almost complete disappearance of probe fluorescence. Caspase-3 can specifically recognize and cleave the peptide substrate at the C-terminus of DEVD to release a FITC-labeled segment (FITC-GDEVD), thus lighting up the fluorescence. In view of the excellent sensitivity and selectivity, the proposed method was further used to evaluate the inhibition efficiency of potential inhibitors and the drug-induced apoptosis of cells.

## 2. Materials and Methods

### 2.1. Chemicals and Reagents

Caspase-3, DEVD-fluoromethylketone (DEVD-FMK), thrombin, and *β*-secretase were obtained from Sigma-Aldrich (Shanghai, China). PQQ, polyvinyl pyrrolidone (PVP), Cu(NO_3_)_2_•3H_2_O, 1,3,5-benzenetricarboxylic acid (BTC), and staurosporine (STS) were purchased from Aladdin Chemistry Co., Ltd. (Shanghai, China). Peptides with the sequence of FITC-GDEVDGHis_6_ and FITC-GDEVD were provided by China Peptides Co., Ltd. (Shanghai, China). Bovine serum albumin (BSA) and Dulbecco’s modified Eagle’s medium (DMEM) were supplied by Sangon Biological Science & Technology Company (Shanghai, China). Prostate-specific antigen (PSA) was supplied by Linc-Bio Science Co., Ltd. (Shanghai, China). All chemical reagents were of analytical grade and used without any purification. Millipore ultrapure water was used for the preparation of all the aqueous solutions.

### 2.2. Apparatus

Fluorescence measurements were carried out on a Hitachi F-4600 fluorescence spectrometer (Hitachi High-Tech, Tokyo, Japan) with an excitation and emission slit width of 5 nm. The morphologies of the Cu-PQQ nanoparticles were captured by a scanning electron microscope (SEM, JSM-IT800, Tokyo, Japan) and an FEI Tecnai G2 T20 transmission electron microscope (TEM, Hillsboro, OR, USA). Element analysis pictures were collected with a Bruker Quantax energy-dispersive X-ray spectroscope (EDS, Berlin, Germany) fitted to the SEM. Dynamic light-scattering measurements were conducted on a Nano ZS90 Zetasizer (Malver Instruments Ltd., Worcestershire, UK). The X-ray photoelectron spectroscopy (XPS) was recorded on an ESCALAB 250Xi instrument (Thermo Scientific, Inc., Waltham, MA, USA). The Fourier transform infrared (FT-IR) spectra were collected on a NEXUS-470 spectrometer (Thermo Scientific, Inc., Waltham, MA, USA).

### 2.3. Fluorescence Titration Experiments

To investigate the binding constant between Cu^2+^ and PQQ in phosphate buffer (10 mM, pH 7.4), fluorescence titration experiments were conducted by monitoring the change in PQQ fluorescence intensity at 487 nm in the presence of different concentrations of Cu^2+^. The binding constant was determined with the Benesi–Hildebrand equation [44]:

$$\frac{1}{F-F_{0}}=\frac{1}{K_{c}\left(F_{s}-F_{0}\right)}+\frac{1}{F_{s}-F_{0}}$$

where F represents the fluorescence intensity of PQQ with different amounts of Cu^2+^ at 487 nm, F_0_ is the fluorescence intensity of PQQ without Cu^2+^, K is the reaction equilibrium constant, c is the concentration of Cu^2+^, and Fs is the fluorescence intensity of PQQ after surfeiting Cu^2+^.

### 2.4. Synthesis of Cu-PQQ Nanoparticles and Cu-BTC MOFs

Cu(NO_3_)_2_ (23 mg) and PQQ (5 mg) were dissolved in 4 mL of DMF (solution A), and PVP (200 mg) was dissolved in 8 mL of DMF–ethanol solution (*v*/*v* = 1:1) (solution B). The dual solutions were mixed and then ultrasonicated for 20 min. Subsequently, the mixed solution was transferred into a Teflon-lined steel autoclave and heated at 100 °C for 8 h. After cooling to room temperature, the precipitates were collected by centrifugation at 5000 rpm/min and washed with 50% ethanol several times. The obtained Cu-PQQ nanoparticles were dried at 60 °C overnight. The stability of Cu-PQQ nanoparticles was investigated by incubating them with Cu^2+^-binding amino acids for 2 h. The changes in fluorescence intensity at 487 nm were monitored.

Cu-BTC MOFs were prepared with the procedure reported in our previous work [50]. In brief, 0.875 g Cu(NO_3_)_2_ and 0.3 g PVP were dissolved in 25 mL DMF. 0.42 g BTC was dissolved in 25 mL DMF. After the two solutions were mixed and stirred for 10 min, the mixture was transferred into a Teflon-lined autoclave and heated at 100 °C for 16 h. The precipitates were centrifugated at 5000 rpm for 10 min and washed with DMF and ethanol several times. The solid products were dried at 60 °C for 12 h under vacuum.

### 2.5. Detection of Caspase-3

For the assay of casapse-3, 100 μL of Cu-PQQ solution (25 μg/mL) was mixed with 100 μL of 2 μM peptide substrate FITC-GDEVDGHis_6_ in phosphate buffer (10 mM, pH 7.4). Then, 50 μL of caspase-3 at a given concentration was added to the mixture and incubated at room temperature for 1 h. Finally, the fluorescence spectra were recorded in the wavelength range of 500–650 nm with an excitation wavelength of 470 nm. For the inhibition evaluation, caspase-3 was pre-incubated with different concentrations of inhibitor for 5 min and then added to the Cu-PQQ/FITC-GDEVDGHis_6_ suspension. The other detection procedures were the same as those for the assay of caspase-3 in the absence of the inhibitor. The inhibition efficiency was determined according to the signal change induced by different concentrations of the inhibitor.

### 2.6. Assays of Caspase-3 in Lysates

Caspase-3 in living and apoptotic cells was extracted according to our previously reported procedure [51]. Briefly, HeLa cells were cultured in DMEM supplemented with 10% fetal bovine serum in an incubator (5% CO_2_, 37 °C) for 24 h. Then, the medium was replaced by the solution containing 10 μM inducer STS. After incubation for 6 h, the cells were collected and washed with the phosphate buffer, followed by treatment with the lysis buffer. Subsequently, 1 mL of the lysate was centrifuged at 13,000 rpm for 10 min at 4 °C. After dilution by different folds with the phosphate buffer, the lysate was added to the Cu-PQQ/FITC-GDEVDGHis_6_ suspension. Other experimental procedures were the same as those mentioned above for the assays of the standard casapse-3 samples.

## 3. Results and Discussion

### 3.1. Characterization of Cu-PQQ Nanoparticles

**Figure 1:**
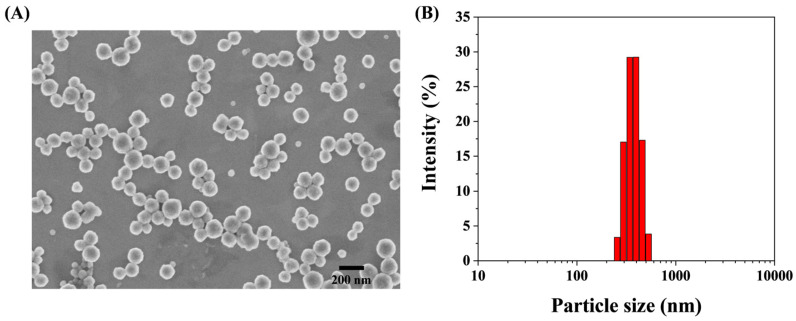

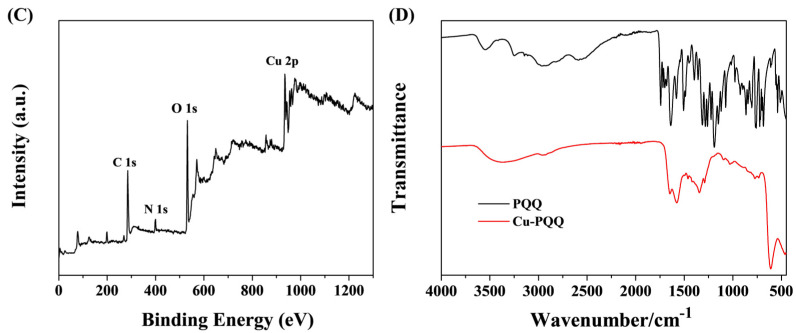
SEM photograph (**A**), size distribution (**B**), XPS survey (**C**), and FT-IR spectrum (**D**) of Cu-PQQ nanoparticles.

Cu-PQQ nanoparticles were synthesized through the coordination interactions between the Cu^2+^ ions and the carboxyl groups in PQQ. The morphology of the synthesized Cu-PQQ nanoparticles was first imaged by SEM and TEM (Figure 1A). Cu-PQQ nanoparticles show a spherical shape, and their average size measured by the dynamic light scattering analyzer is about 540 nm (Figure 1B). The XPS spectra confirmed the chemical components of C, O, Cu, and N elements in the nanoparticles (Figure 1C). The Cu 2p of Cu-PQQ can be decomposed into two peaks of 954.82 eV and 934.79 eV (Figure S1A), which are matched with Cu 2p1/2 and Cu 2p3/2, respectively. The deconvoluted peaks at 941.00, 943.56, and 962.75 eV are assigned to the satellite peaks. These results illustrate that the Cu element in the oxo-node of Cu-PQQ is mainly in Cu(II) state. The O 1s spectrum for Cu-PQQ presented two peaks (Figure S1B), which corresponded to 532.50 eV (C–OH) and 531.40 eV (C=O). In addition, the Fourier transform infrared (FT-IR) spectra confirmed the coordination of Cu^2+^ ions with the carboxyl groups in PQQ. As shown in Figure 1D, after complexation with Cu^2+^, the relative intensity of PQQ in stretching vibration peak from O–H and H bonding in the band of 2500~3000 cm^−1^ decreased, indicating that Cu^2+^ was coordinated by the carboxyl groups of PQQ. However, no ordered structure crystals were formed for Cu-PQQ because no characteristic diffraction peaks were found in the XRD spectrum range of 10–25° (Figure S2). Thus, the nanoparticles were defined as metal–organic hybrids but not frameworks in this work.

### 3.2. Stability of Cu-PQQ Nanoparticles

**Figure 2:**
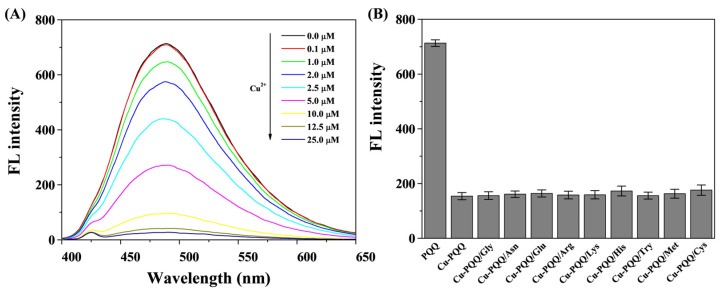
(**A**) The fluorescence spectra of 5 μM PQQ in the absence and presence of different concentrations of Cu^2+^. (**B**) The fluorescence intensity of 0.01 mg/mL Cu-PQQ nanoparticles in the absence and presence of various amino acids at a concentration of 50 μM. The excitation wavelength was 340 nm.

To investigate the binding constant between Cu^2+^ and PQQ, fluorescence titration assays were conducted by monitoring the change fluorescence intensity of PQQ in the presence of different concentrations of Cu^2+^ (Figure 2A). The binding constant was found to be 6.2 × 10^4^ M^−1^ based on the Benesi–Hildebrand equation [44]. The stability of the Cu-PQQ nanoparticles was investigated by incubation with several natural amino acids. No significant change in the fluorescence intensity at 487 nm was observed (Figure 2B), indicating that the coordinated Cu^2+^ could not be sequestrated by the tested amino acids. The high stability of the Cu-PQQ nanoparticles can be attributed to the strong coordination interactions of the carboxyl groups and the aromatic nitrogens in PQQ to Cu^2+^ [44,52].

### 3.3. Quenching Efficiency

**Figure 3:**
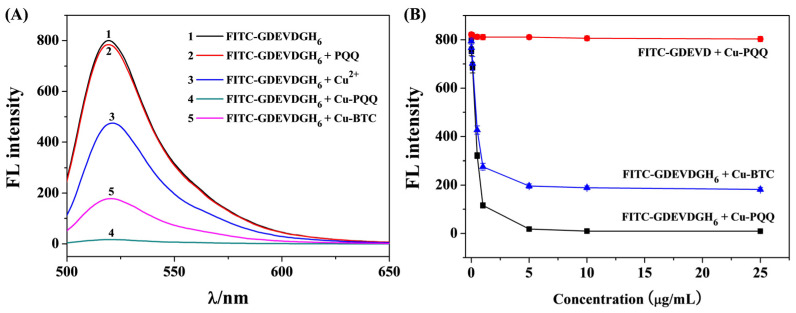
(**A**) The fluorescence spectra of 1 μM FITC-GDEVDGH_6_ in the absence (curve 1) and presence of PQQ (curve 2), Cu^2+^ (curve 3), 10 μg/mL Cu-PQQ nanoparticles (curve 4), and 10 μg/mL Cu-BTC MOFs (curve 5). (**B**) The effect of Cu-PQQ and Cu-BTC dosage on the fluorescence intensity of an FITC-labeled peptide.

Cu-PQQ was composed of dual quenching cofactors (Cu^2+^ and PQQ) that could quench the fluorescence of dyes by electron and energy transfer effects. A FITC-labeled peptide containing a DEVD sequence specific to caspase-3 (FITC-GDEVDGH_6_) was selected as the substrate. The fluorescence spectra of FITC-GDEVDGH_6_ in different solutions were collected. As shown in Figure 3A, no significant change in the fluorescence intensity was observed after the addition of PQQ (curves 1 and 2), indicating that there is no interaction between PQQ and the peptide probe. In the presence of Cu^2+^, the fluorescence was quenched by ~40% (curve 3), which is attributed to the paramagnetic Cu^2+^-triggered photoinduced electron transfer (PET) process [47]. The addition of Cu-PQQ caused a significant decrease in the fluorescence intensity (curve 4), demonstrating that Cu-PQQ could capture the probe and effectively quench its fluorescence. To prove the role of PQQ in the quenching process, other metal–organic hybrids such as Cu-BTC MOFs with a single quenching cofactor (Cu^2+^) were employed to quench the fluorescence of FITC-GDEVDGH_6_. Consequently, a relatively small decrease in the fluorescence intensity was observed for Cu-BTC (curve 5), suggesting that the dye can be quenched by the d^9^ metal center Cu^2+^ via the PET effect [53,54]. To further gain insight into the quenching ability of Cu-PQQ, the effect of Cu-PQQ and Cu-BTC dosages ranging from 0 to 25 μg/mL on the fluorescence of FITC-GDEVDGH_6_ was studied. As shown in Figure 3B, the fluorescence intensity decreased sharply along with the increment of the Cu-PQQ dosage and almost reached the background level. The quenching efficiencies of Cu-PQQ and Cu-BTC were found to be 98% and 77%, respectively, indicating that the metal–organic hybrids with dual quenching cofactors exhibited higher quenching ability than those with a single quenching cofactor. In addition, we found that Cu-PQQ exhibited a negligible fluorescence quenching effect on the His_6_-free peptide FITC-GDEVD. The result indicated that the His_6_-tagged peptide probe could be attached onto the surface of Cu-PQQ by the metal coordination interactions. We also found that Cu-PQQ exhibited a high fluorescence quenching effect even after storage at 4 °C for three months.

### 3.4. Sensitivity for Caspase-3 Detection

**Figure 4:**
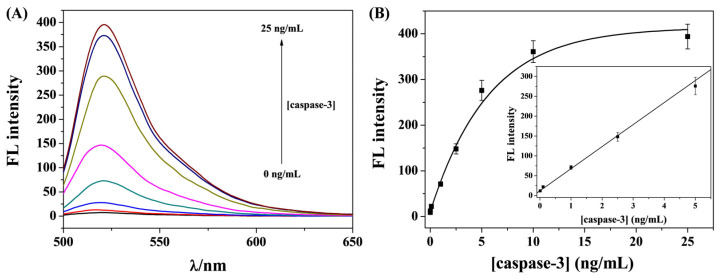
(**A**) Fluorescence spectra and (**B**) calibration plots for the detection of different concentrations of caspase-3 (0, 0.01, 0.1, 1, 2.5, 5, 10, and 25 ng/mL). The inset in panel (**B**) shows the linear portion of the fitting curve.

In this work, caspase-3 was determined as the model protease. The peptide substrates (FITC-GDEVDGH_6_) were anchored onto the Cu-PQQ by the metal coordination interactions between the His_6_ tags and the unsaturated Cu^2+^ ions. Caspase-3 can specifically recognize and cleave the peptide substrate at the C-terminus of DEVD. The feasibility and sensitivity of this method were studied by exposing the FITC-GDEVDGH_6_/Cu-PQQ nanoprobe to a series of concentrations of caspase-3 for a given time. The fluorescence emission spectra were collected and are presented in Figure 4A. The fluorescence intensity increased gradually with the increase in caspase-3 concentration from 0 to 25 ng/mL. This indicates that a higher concentration of caspase-3 can catalyze the cleavage of more peptide probes, thus resulting in the release of more FITC-GDEVD fragments from the Cu-PQQ surface. According to the recovery of the emission signal, a good linear relationship between the fluorescence intensity and the caspase-3 concentration was achieved in the range from 0.01 to 5 ng/mL (Figure 4B). The regression equation can be expressed as *F* = 55.5[caspase-3] + 12.5 (*R*^2^ = 0.986), where *F* refers to the fluorescence intensity at 520 nm and [caspase-3] refers to the concentration of caspase-3. The detection limit was found to be 7 pg/mL based on the 3δ/slope rule. The value was comparable to or even much lower than that of other fluorescence assays achieved with gold nanostructures, carbon nanomaterials, polymers, MoS_2_ nanosheets, and MOFs as the quenchers (Table 1). The high sensitivity can be attributed to the low background signal of the nanoprobe, the powerful immobilization of the His_6_-tagged peptide substrate on the nanoparticle surface, and the enhanced quenching efficiency. In addition, the method for probe immobilization on the surface of Cu-PQQ is simple and powerful, which can facilitate the application of metal–organic hybrids for the rapid determination of protease activity.

### 3.5. Selectivity

**Figure 5:**
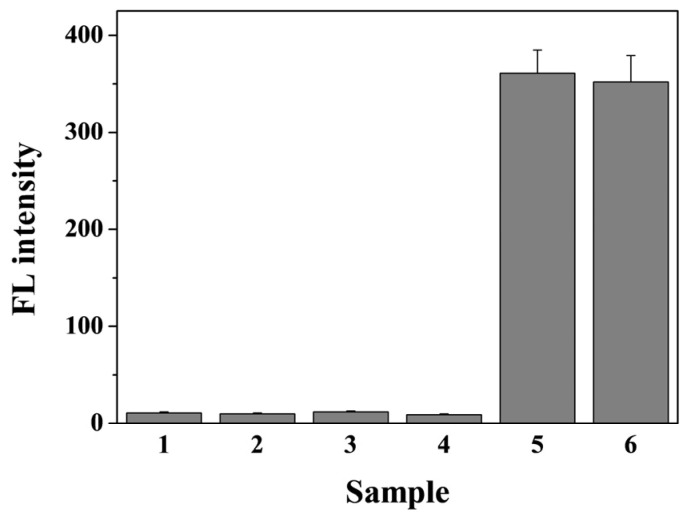
The selectivity of the nanoprobe (from bar 1 to 6: BSA, trypsin, PSA, *β*-secretase, caspase-3, and the mixture of 1~6). The concentration of caspase-3 was 10 ng/mL and that of the others was 50 ng/mL.

Selectivity is an important issue in assessing the performance of analytical methods. The selectivity of the nanoprobe for caspase-3 detection was examined by challenging it with serum protein BSA and three non-specific proteases (trypsin, PSA, and *β*-secretase). As shown in Figure 5, no obvious increase in the fluorescence intensity was observed for the tested interferences (bars 1~4). In addition, there is no significant difference in the fluorescence intensity for the detection of caspase-3 in the absence and presence of the tested interfering species (bars 5~6). This indicated that the peptide probe was attached onto the nanoquencher surface with a high degree of stability, and it could not be replaced by the interfering proteins. We have also found that metal ions in biological systems (e.g., Ca^2+^, Mg^2+^, Fe^2+^, Zn^2+^, and Co^3+^) did not induce significant changes in the fluorescence intensity of nanoprobes (Figure S3). The good selectivity of this method could be attributed to the high specificity of caspase-3 toward the substrate, the strong coordination interaction between His_6_ tags and metal ions, and the negligible quenching effect of Cu-PQQ on the released FITC-included segment.

### 3.6. Inhibition Efficiency

**Figure 6:**
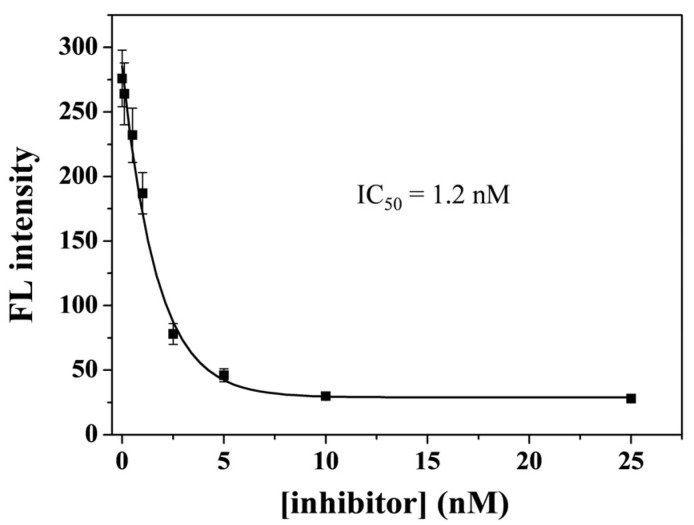
Dependence of fluorescence intensity on inhibitor concentration with 5 ng/mL of caspase-3.

Given that caspase-3 is believed to be a credible target for the treatment of apoptosis-related diseases, the screening of its inhibitors is of great importance to the pharmaceutical industry. In this work, DEVD-FMK was selected as a model inhibitor to prove the feasibility of this method for screening potential inhibitors. Caspase-3 (5 ng/mL) was treated with different concentrations of DEVD-FMK and then analyzed by the proposed nanoprobe. As presented in Figure 6, the fluorescence intensity decreased gradually with the increase in DEVD-FMK concentration. This result indicates that inhibiting the activity of caspase-3 by DEVD-FMK could effectively prevent the cleavage of a peptide substrate, thus limiting the release of a FITC-included segment from the nanoquencher surface. The half-maximum inhibition value (IC_50_) was estimated to be 1.2 nM, which is consistent with that found by other assays [70,71]. Therefore, the method shows great potential in the rapid and high-throughput screening of drugs to inhibit protease activity.

### 3.7. Assays of Caspase-3 Activity in Cell Lysates

**Figure 7:**
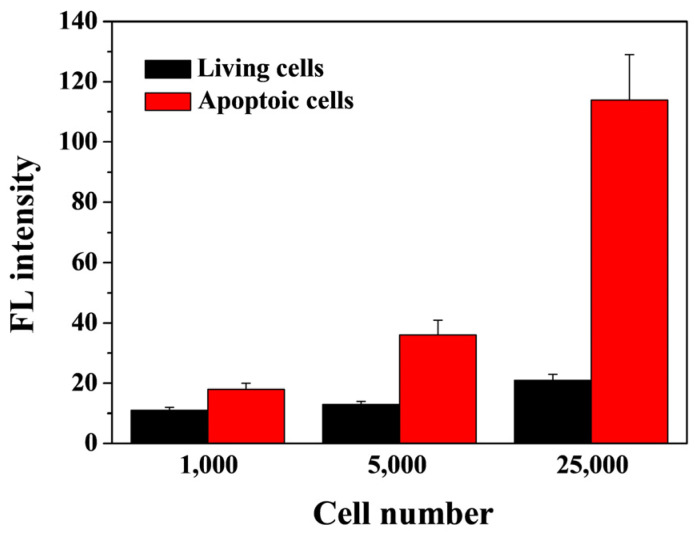
Dependence of fluorescence intensity on cell number.

Caspase-3 is considered to be a key enzyme for the execution of cell apoptosis. Therefore, the sensitive detection of caspase-3 is of great importance for the diagnosis and prognosis of apoptosis-relative diseases. In order to evaluate the feasibility of this method for application in biological systems, the activity of caspase-3 in living and apoptotic cancer cells was determined. STS was used as the inducer to induce the apoptosis of HeLa cells, and then the lysates were extracted for in vitro assays of caspase-3. There was no significant fluorescence recovery when the nanoprobe was incubated with the lysate extracted from the living cells (black bars) (Figure 7). However, the fluorescence intensity was greatly intensified for the lysate extracted from the apoptotic cells (red bars), and the value was obviously higher than that of the living cells. We also found that the addition of inhibitor DEVD-FMK to the lysate extracted from the apoptotic cells could limit the fluorescence enhancement, indicating that caspase-3 was activated during the apoptosis of HeLa cells and the method showed high specificity for the assay of active caspase-3.

## 4. Conclusions

In conclusion, a new metal–organic hybrid with dual quenching cofactors (Cu^2+^ and PQQ) was prepared and used as the nanoquencher for the fluorescence sensing of protease activity. The hybrid exhibited higher quenching efficiency than that composed of a single quenching cofactor. In addition, the procedure for substrate immobilization on the nanoquencher surface by the metal–His_6_ coordination interaction was simple and time-saving, and the method required the use of a single labeled fluorescence probe. It achieved the detection of caspase-3 with a low background signal and high sensitivity. Furthermore, the nanoprobe was used to monitor caspase-3 activity, determine inhibition efficiency, and evaluate cell apoptosis with satisfactory results. The method should be useful for the design of novel nanoquenchers and the development of other protease biosensors by changing the quenching cofactors and peptide sequences.

## Figures and Tables

**Table 1:** An overview of the fluorescence assays of caspase-3 with different nanomaterials as the quenchers.

Quencher	Fluorophore	Linear Range	Detection Limit	Ref.
AuNPs	5-TAMRA	0–1 unit/mL	0.0079 unit/mL	[55]
AuNPs	5-TAMRA	0–300 ng/mL	73 pg/mL	[56]
AuNP	FAM	10–300 ng/mL	10 ng/mL	[57]
AuNPs	CdTe QDs	27.68–129.15 pM	18 pM	[58]
AuNPs	Ru@SiNPs	0.05–1 U/mL	0.05 U/mL	[59]
Au NBPs@PDA	FITC	0–100 ng/mL	99 pg/mL	[60]
AuNPs@Mem	FITC	3.2–100 pg/mL	1.3 pg/mL	[61]
GO	FAM	7.25–362 ng/mL	7.25 ng/mL	[62]
GO	FAM	0.5–6.0 U/mL	0.5 U/mL	[63]
rGO	FAM	0.4–7 U/mL	0.33 U/mL	[64]
MCN	FITC	0.01–1 ng/mL	0.4 pg/mL	[65]
MOFs	AuNCs	0.3–150 ng/mL	0.12 ng/mL	[66]
MoS_2_@PDA	FAM	2–360 ng/mL	0.33 ng/mL	[67]
PNPs	FAM	2–40 nM	0.09 nM	[68]
UCNPs	RB	0.01–1000 pg/mL	0.01 pg/mL	[69]
Cu-PQQ	FITC	0.01–5 ng/mL	7 pg/mL	This work

Abbreviations: AuNPs, gold nanoparticles; QDs, quantum dots; Ru@SiNPs, [Ru(bpy)_3_]^2+^, encapsulated silica nanoparticles; Au NBPs, gold nanobipyramids; PDA, polydopamine; AuNPs@Mem, membrane-functionalized gold nanoparticles; GO, graphene oxide; rGO, reduced graphene oxide; MCN, mesoporous carbon nanospheres; PNPs, polymeric nanoquenchers; MoS_2_, MoS_2_ nanosheets; MOFs, metal–organic frameworks; AuNCs, gold nanoclusters; PNPs, polymeric nanoquenchers; UCNPs, NaGdF_4_:Yb^3+^, Er^3+^@NaGdF_4_ upconversion nanoparticles; RB, Rhodamine B.

## Data Availability

The original contributions presented in this study are included in the article. Further inquiries can be directed to the corresponding author(s).

## Supplementary Materials

The following supporting information can be downloaded at: https://www.mdpi.com/article/10.3390/bios15060354/s1. Figure S1. High-resolution spectra of Cu 2p and O 1s of the synthesized Cu-PQQ nanoparticles. Figure S2. XRD pattern of Cu-PQQ nanoparticles. **Figure S3**. Selectivity of the nanoprobe in the presence of Ca^2+^, Mg^2+^, Fe^2+^, Zn^2+^, and Co^3+^.
