# Supplementary material for: A Metal–Organic Hybrid Composed of Dual Quenching Cofactors as a Nanoquencher for the Fluorescent Determination of Protease Caspase-3
Source: Biosensors (Basel). 2025 Jun 4;15(6):354. doi: 10.3390/bios15060354 (PMC12190246; doi:10.3390/bios15060354)
Supplement: Supplementary file 1 [file biosensors-15-00354-s001.zip › biosensors-3601037-supplementary.pdf]

A Metal-Organic Hybrid Composing of Dual Quenching Cofactors as the Nanoquencher for Fluorescent Determination of Protease Caspase-3

Fengli Gao [1], Lin Liu [1,*], Cancan He [1], Yong Chang [1,2,*] and Weiqiang Wang [3]

[1] Henan Province Key Laboratory of New Opto-electronic Functional Materials, College of Chemistry and Chemical Engineering, Anyang Normal University, Anyang, Henan 455000, China

[2] Shiyan Key Laboratory of Biological Resources and Eco-environmental Protection, Department of Chemistry and Environmental Engineering, Hanjiang Normal University, Shiyan, 442000, China

[3] International Joint Laboratory of Henan Photoelectric Functional Materials, College of Chemistry and Chemical Engineering, Anyang Normal University, Anyang, Henan 455000, China

*Correspondence: liulin@aynu.edu.cn (L.L.); yongchang_swudc@163.com (Y.C.)

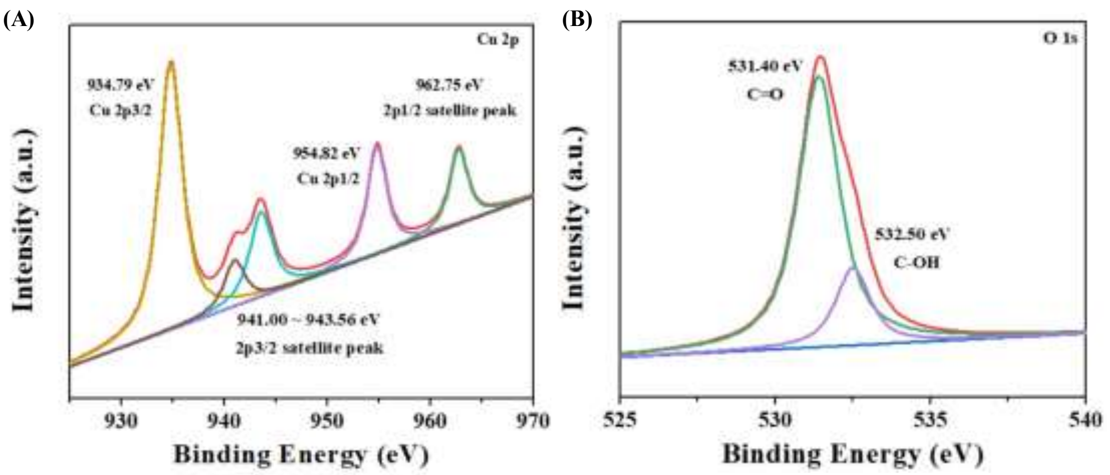

Figure S1. High-resolution spectra of (**A**) Cu 2p and (**B**) O 1s of the synthesized Cu-PQQ nanoparticles.

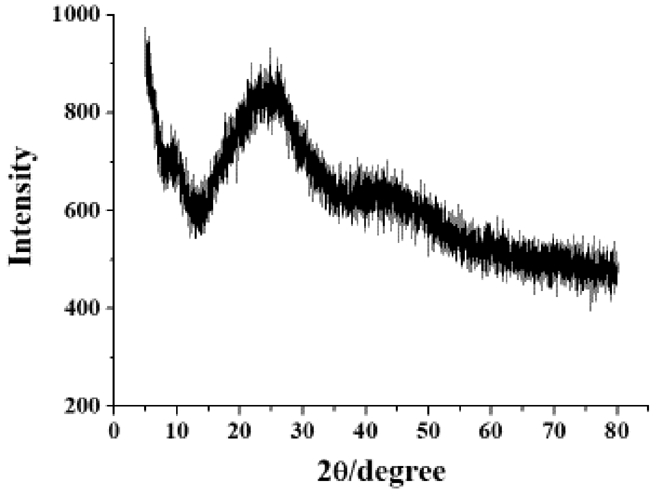

Figure S2. XRD pattern of Cu-PQQ nanoparticles.

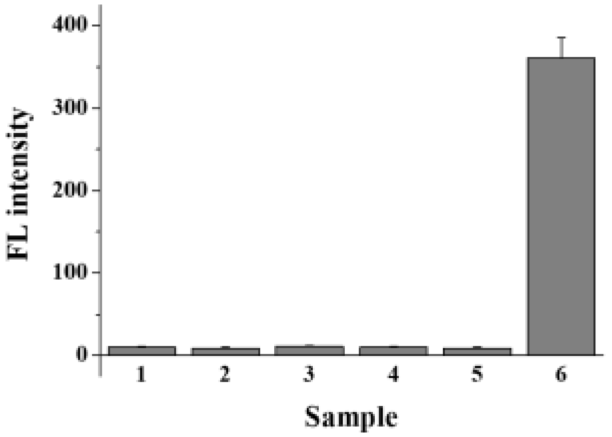

Figure S3. Selectivity of the nanoprobe in the presence of Ca^{2+} (bar 1), Mg^{2+} (bar 2), Fe^{2+} (bar 3), Zn^{2+} (bar 4), Co^{3+} (bar 5), and caspase-3 (bar 6). The concentration of caspase-3 was 10 ng/mL and that of metal ions was 0. 1 mM.
